# GH responsiveness in a large multinational cohort of SGA children with short stature (NESTEGG) is related to the exon 3 GHR polymorphism

**DOI:** 10.1111/j.1365-2265.2007.02911.x

**Published:** 2007-09

**Authors:** M Tauber, W Ester, F Auriol, C Molinas, J Fauvel, J Caliebe, T Nugent, L Fryklund, M B Ranke, M O Savage, A J L Clark, L B Johnston, A C S Hokken-Koelega

**Affiliations:** * Division of Endocrinology, Genetics, Gynaecology and Bone Diseases, Hôpital des Enfants Toulouse, France; † Unité INSERM U563 (CPTP), IFR 30, Hôpital Purpan Toulouse, France; ‡ Department of Paediatrics, Division of Endocrinology, Erasmus MC-Sophia Children's Hospital Rotterdam, the Netherlands; § Laboratoire de Biochimie 3, Institut Fédératif de Biologie, Hôpital Purpan Toulouse, France; ¶ Paediatric Endocrinology Section, University Children's Hospital Tuebingen, Germany; ** Pfizer Health AB Sollentuna, Sweden; †† Department of Endocrinology, Barts and the London Queen Mary School of Medicine, John Vane Science Centre London, UK

## Abstract

**Objective:**

The polymorphic deletion of exon 3 of the GH receptor (d3-GHR) has recently been linked to the magnitude of growth response to recombinant human GH (rhGH) therapy in short children with or without GH deficiency. We investigated this association in a large multinational cohort from the Network of European Studies of Genes in Growth (NESTEGG), comprising short children born small for gestational age (SGA).

**Design:**

The study included short prepubertal SGA children treated with rhGH for 1 or 2 years.

**Population:**

Two hundred and forty white Caucasian SGA children (138 male, 102 female) aged 6·6 ± 2·3 years with a height at –3·0 ± 0·7 SDS at start of rhGH treatment; 193 ethnically matched controls.

**Methods:**

The GHR polymorphism (fl/fl, fl/d3 or d3/d3) was genotyped by polymerase chain reaction (PCR) multiplex assay. Growth velocity (G/V) in cm/year and changes in GV during the first and second year of rhGH treatment were evaluated.

**Results:**

The change in GV was significantly greater in SGA children carrying one or two copies of the d3-GHR allele (*P* = 0·038 for the first year and *P* = 0·041 for the second year of GH treatment), but the change in height was not significantly different. Birthweight was significantly lower in SGA children with the d3/d3 genotype than in SGA children with the fl/fl genotype (*P* = 0·034) and in those with the fl/d3 genotype (*P* = 0·016).

**Conclusion:**

Our data, based on a large cohort, showed that the exon 3 GHR polymorphism is associated with responsiveness to rhGH treatment in SGA children with short stature.

## Introduction

Response to recombinant human growth hormone (rhGH) therapy in short children without GH deficiency is variable^1^, ^2^ and the prediction of this response may not be accurate. Prediction models based on variables such as age, weight, parental height and rhGH dosage only explain half of the response to rhGH, suggesting the existence of genetic and environmental determinants still unknown.^3^ GH acts at the target cell through the GH receptor, which, after binding of the ligand, stimulates a signalling cascade that leads to target genes transcription. This receptor contains an unusual genetic polymorphism caused by a genomic deletion of exon 3 (d3-GHR) that mimics alternative splicing.^4^ This deletion results in the loss of amino acid residues 7–28 and the amino acid substitution A6D at the N-terminal part of the extracellular domain, although the deleted protein does not have altered affinity for GH.^5^ It has been demonstrated, in transfection experiments, that the transduction of GH signalling through d3-GHR homo- or heterodimers was almost 30% higher than through the full-length GHR homodimer.^6^ These data corroborate the association of the d3-GHR variant with increased responsiveness to rhGH in short children without GH deficiency. The children genotyped in this first report consisted of short children born small for gestational age (SGA) (*n* = 60) and with idiopathic short stature (*n* = 112).^6^ Another publication has confirmed the increased rhGH responsiveness in Turner syndrome (*n* = 53) and SGA (*n* = 60), 7 but a recent study of short children born SGA did not confirm these data.^8^ We have investigated this association in the cohort from the Network of European Studies of Genes in Growth (NESTEGG), 9 consisting of children from four different countries (France, the Netherlands, the UK and Germany).

## Subjects and methods

### Patients

The NESTEGG project, which had been approved in all countries by their own ethical committees, recruited children born SGA, children with idiopathic short stature and controls across four different countries (France, the Netherlands, the UK and Germany) according to a standard protocol where detailed phenotypic features were recorded and blood sampling was undertaken to provide DNA.^9^

Prepubertal white Caucasian SGA children (*n* = 240) with short stature (height SDS ≤ –1·88 and/or weight SDS ≤ –1·88) treated with rhGH for at least 1 year were included in the present analysis. We chose the cut-off of –1·88 instead of –2 SDS to avoid the exclusion of some borderline children. Exclusion criteria were a known syndrome or dysmorphic features, known chromosomal or genetic abnormality, known severe chronic illness or endocrine disease or positive gliadin, endomysial or reticulin antibodies, and severe disproportionate short stature.

All children were evaluated at baseline and every 3 months during rhGH treatment. Evaluations included measurements of weight (using a digital scale), standing height (standing height was the mean of three measurements with a Harpenden stadiometer) and pubertal status graded according to Marshall and Tanner.^10^, ^11^ One hundred and ninety children were treated for 2 years. The phenotypic characteristics are presented in Table 1. The sex ratio was 138 boys to 102 girls with a mean birth length of –2·9 ± 1·4 SDS and birthweight of –2·3 ± 1·0 SDS. Mean maternal height was –1·0 ± 1·3 SDS and mean paternal height –0·8 ± 1·0 SDS. Age at onset of rhGH treatment was 6·6 ± 2·3 years with a mean height of –3·0 ± 0·7 SDS and a mean weight of –2·4 ± 0·9 SDS. The mean rhGH dose was 0·4 ± 0·17 mg/kg/week. Forty-three patients received a replacement rhGH dose (0·21 ± 0·04 mg/kg/week) while the majority of children (*n* = 197) received a higher dose of rhGH (0·43 ± 0·17 mg/kg/week).

**Table 1:** Phenotype and genotype of NESTEGG short SGA (*n* = 240) and controls (*n* = 193). Data are presented as mean ± SD or percentage

	SGA (*n* = 240)	Controls (*n* = 193)
*Phenotype*		
Sex ratio (M/F)	138/102	
Birth length (SDS)	–2·9 ± 1·4	
Birthweight (SDS)	–2·3 ± 1·0	
Maternal height (SDS)	–1·0 ± 1·3	
Paternal height (SDS)	–0·8 ± 1·0	
Age at onset of GH treatment (years)	6·6 ± 2·3	
Height at start of GH treatment (SDS)	–3·0 ± 0·7	
Weight at start of GH treatment (SDS)	–2·4 ± 0·9	
GH dose (mg/kg/week)	0·40 ± 0·17	
*Genotype*		
fl/fl, % (*n*)	60 (144)	51 (98)
fl/d3, % (*n*)	27 (65)	41 (80)
d3/d3, % (*n*)	13 (31)	8 (15)

### Growth prediction

For each child, a growth prediction for the first year of therapy was calculated according to Ranke *et al*.^2^ The prediction model incorporates rhGH dose, age and weight at start of therapy (given in SDS according to Freeman *et al*.^12^) as well as gender-adjusted midparental height (MPH) (in SGA) or the distance to MPH (given in height SDS according to Tanner *et al*.^13^, ^14^) as the main prognostic factors. The equation for prediction in short SGA children is: growth during the first year (cm) = 9·4 + [56·51 × rhGH dose (mg/kg/day)] + [–0·31 × age at onset (years)] + [0·30 × body weight SDS at start] + [0·11 × MPH SDS].

The individual deviations from the prediction were divided by the standard error of the prediction, which is 1·30 cm in SGA children, 2 resulting in the studentized residuals. A studentized residual of 0 means that the prediction was completely fulfilled. Positive values indicate growth exceeding the prediction, negative values are the expression of growth slower than predicted.

### Controls

The controls were of white Caucasian descent and were recruited in early to mid-adulthood according to a standard protocol, where auxological details (birth length, birthweight, gestational age, current weight and height, blood pressure, current medical history and medication) were recorded and a blood sample was drawn for DNA extraction.

### Molecular studies

Genomic DNA was isolated from peripheral blood leucocytes by standard methods from all patients. The frequency of the GHR transcript variant [full-length (fl) or exon 3 deleted (d3)] was tested in all subjects using a simple multiplex polymerase chain reaction (PCR) assay described by Pantel *et al*.^4^ Amplification products were analysed by electrophoresis on a 1% agarose gel stained with ethidium bromide. The fl-GHR is represented by a 935-bp fragment and the d3-GHR by a 532-bp fragment.

### Statistical analysis

Qualitative variables are listed as frequencies and percentages, whereas quantitative variables are shown as mean ± SD, or median and range. Patients were divided by genotype and compared with regard to continuous parameters (e.g. age, midparental height in SDS, height at start of treatment in SDS, birth length and birthweight in SDS, paternal and maternal height in SDS), as well as changes in height velocity.

The response to rhGH was evaluated by annual growth velocities (in cm/year) and by the differences between growth velocity before GH treatment and during the first and/or second year of GH treatment as published.^6^, ^7^ Height velocity expressed in SDS was not chosen as all children were prepubertal and there was no sex difference at this age.

The d3/d3 and d3/fl patients were grouped together (group D) to compare them with patients with the fl/fl genotype.

Comparisons between groups were made by the Mann–Whitney test or the Kruskall–Wallis test, as appropriate. A *P*-value of less than 0·05 was considered statistically significant. All statistical analyses were performed with Statview for Windows.

## Results

### Distribution of d3-GHR and fl-GHR

The calculated allele frequencies were 73·5% for fl-GHR *vs.* 26·5% for d3-GHR in the SGA population and 73% *vs.* 27% in the controls. Table 1 shows the distribution of the genotypes in the SGA children and in the controls.

### GHR exon 3 genotypes and clinical characteristics

In the SGA children, the mean birthweight in SDS was significantly lower in the d3/d3 group than in the two other groups: –2·6 ± 0·7 SDS *vs.* –2·2 ± 0·9 SDS in the fl/d3 group (*P* = 0·016) and –2·2 ± 0·7 SDS in the fl/fl group (*P* = 0·034). This difference was not present in the control population: –0·5 ± 0·8 SDS in the d3/d3 group *vs.* –0·3 ± 1·0 in the fl/d3 group and –0·4 ± 1·0 in the fl/fl group.

There was no significant difference in birth length, age, height and weight at start of GH treatment, parental heights and spontaneous height velocity between the three different genotype groups.

### GHR exon 3 genotypes and response to rhGH in short SGA children

**Fig. 1:**
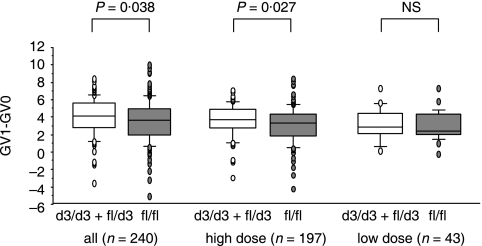
Change in growth velocity during the first year of GH treatment in the 240 SGA children according to the dose of rhGH: high dose 0·43 ± 0·17 mg/kg/week; low dose 0·21 ± 0·04 mg/kg/week. Results are presented as boxes with the horizontal line inside the box representing the mean and the vertical line representing the interquartile range (10th–90th centiles). The extreme values are presented as circles. The d3/d3 + fl/d3 group (or D group) is in white and the fl/fl group is in grey.

The change in height velocity during the first and the second year was not significantly different between the three groups. However, the combined group of d3/d3 and d3/fl children (group D) showed a significantly higher change of growth velocity during the first year (*P* = 0·038, Fig. 1), second year (0·041) and both years (*P* = 0·03). Data are shown in detail in Table 2.

**Table 2:** Growth velocities (cm/year) and changes in growth velocities before and during the first 2 years of GH treatment. Data are presented as median (range)

	d3/d3 (*n* = 31)	fl/d3 (*n* = 65)	fl/fl (*n* = 144)	(fl/d3 + d3/d3) or D (*n* = 96)
GV0	5·3 (3·0–11·0)	5·4 (2·0–11·2)	5·6 (0·8–13·1)	5·3 (2·0–11·2)
GV1	9·9 (5·8–13·5)	9·3 (5·9–14·3)	9·3 (4·9–15·0)	9·6 (5·8–14·3)
GV2	7·5 (4·0–10·6)	8·0 (5·3–12·1)	7·7 (3·6–10·8)	7·9 (4·0–12·1)
(GV1 + GV2)/2	9·1 (4·9–12·0)	8·8 (6·7–13·2)	8·7 (4·9–12·3)	8·9 (4·9–13·2)
Δ(GV1 – GV0)	4·4 (–3·6 to 8·4)	4·1 (–1·4 to 7·6)	3·7 (–5·1 to 10·0)	4·2 (–3·6 to 8·4)*
Δ(GV2 – GV0)	2·5 (–1·8 to 6·0)	2·6 (–2·7 to 7·0)	2·1 (–6·1 to 7·4)	2·6 (–2·7 to 7·0)†
Δ[(GV1 + GV2)/2 – GV0]	3·4 (–0·3 to 6·9)	3·2 (–1·9 to 6·4)	2·8 (–4·6 to 8·5)	3·2 (–1·9 to 6·9)‡
Studentized residuals GV1	0·25 (–2·8 to 2·2)	0·02 (–2·5 to 3·5)	–0·20 (–3·6 to 2·8)§	0·03 (–2·8 to 3·5)

D, the combination of the two groups fl/d3 and d3/d3; GV0, growth velocity before GH treatment; GV1, growth velocity during the first year of GH treatment; GV2, growth velocity during the second year of GH treatment; (GV1 + GV2)/2, mean growth velocity during the first 2 years of GH treatment.

* *P* = 0·038 between fl/fl and D groups.

† *P* = 0·041 between fl/fl and D groups.

‡ *P* = 0·03 between fl/fl and D groups.

§ *P* = 0·04 indicates significant difference between observed and predicted growth velocity using the SGA prediction model.

When we divided the children into two groups according to rhGH dose [high dose (*n* = 197) and low dose (*n* = 43)], a significant change in growth velocity during the first year was observed in the high-dose group (*P* = 0·027) but not in the low-dose group (Fig. 1).

**Fig. 2:**
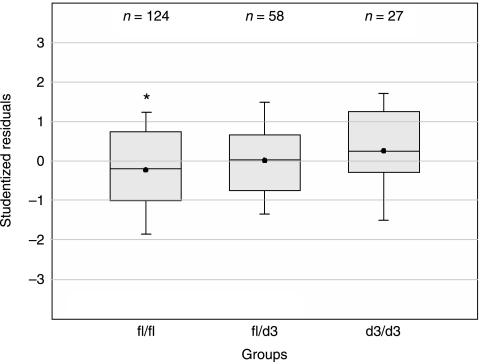
Studentized residuals in the three genotype groups are calculated according to the KIGS growth prediction model for SGA, 2 incorporating rhGH dose, age and weight at start of therapy as well as gender-adjusted midparental height. Results are presented as boxes with the horizontal line inside the box representing the mean and the vertical line representing the interquartile range (10th–90th centiles). Positive studentized residuals indicate growth that exceeded the prediction, negative values growth below the prediction. The asterisk indicates a significant difference between the predicted growth velocity and the observed growth velocity (*P* = 0·04).

Studentized residuals in the fl/fl group were significantly lower (*P* = 0·04), meaning that these children grew less than predicted, while they was no significant difference in the other two groups (Fig. 2).

## Discussion

We took the opportunity of the NESTEGG project to analyse a subgroup of this population with prepubertal short SGA children treated with rhGH for 1 or 2 years.

This study, of a large multinational cohort of short SGA children, is unique and showed the association of the exon 3 GHR polymorphism with rhGH responsiveness in the largest population reported so far. In the first study, 6 performed in France, SGA children represented 35% of the study population (60 SGA among 172 short children). Another study was performed in Germany in 60 SGA children.^7^ In both studies the exon 3 GHR polymorphism was associated with rhGH responsiveness. Conversely, a more recent publication did not report any significant difference in GH responsiveness in a large Spanish population of 170 SGA children and adolescents.^8^

We did not find a significantly higher growth velocity in the d3/d3 group as published.^7^ Nevertheless, we did find a significant larger change in growth velocity, only in the mixed group of homozygotes and heterozygotes, during the first year and the first 2 years of GH treatment in this population of short SGA children, as reported in the first study.^6^ This suggests the involvement of other factors. In another study combining different populations with short stature^7^ (Turner and SGA), the effect of this polymorphism in short SGA children was lower than in GH-deficient^15^ or Turner^7^ children in terms of growth velocity. The influence of the exon 3 GHR polymorphism in SGA became more evident when growth was analysed by the SGA growth prediction model, which takes into consideration the variability of the response to treatment between individuals. The magnitude of the difference is small but in the same range as in the previous publication.^7^

In our study, the association of the exon 3 GHR polymorphism with growth responsiveness was observed in the group with the higher rhGH dose (0·43 ± 0·17 mg/kg/week). The lack of significance in the group with a lower dose could be due to the small number of children (43 *vs.* 197).

Discordant results regarding the exon 3 GHR polymorphism and rhGH responsiveness have also been reported a in GH-deficient population.^15^, ^16^ The NESTEGG cohort is a multinational cohort, which could reduce the effect of specific population polymorphisms. The distribution of the three genotypes is not significantly different in this study compared to the different reports in Caucasian populations.^6^, ^7^, ^16^

In our study, the frequency of the d3-GHR variant was comparable in the SGA and in the controls, suggesting that this polymorphism is not primarily related to the genesis of SGA. However, a recent publication^17^ performed in a Spanish population reported a significantly higher frequency of the fl/fl genotype in SGA compared to controls.

In our study, birthweight was significantly lower in the d3/d3 group, while this difference was not observed in the controls. As the expression of the GHR is low before birth, it is unlikely that this difference is due to the exon 3 GHR polymorphism.

Our data, based on a large cohort, showed that the exon 3 GHR polymorphism is associated with the responsiveness to rhGH treatment in SGA children with short stature. However, given the contradictory findings in other reports, this polymorphism is probably not a major factor among those influencing GH response.
